# Supplementary material for: Weak phases production and heat generation control fault friction during seismic slip
Source: Nat Commun. 2020 Jan 17;11:350. doi: 10.1038/s41467-019-14252-5 (PMC6969095; doi:10.1038/s41467-019-14252-5)
Supplement: Supplementary file 1 — Supplementary Information [file 41467_2019_14252_MOESM1_ESM.pdf]

Supplementary information for: Weak phases production and heat generation control fault friction during seismic slip

by Rattez and Veveakis

Supplementary Figures

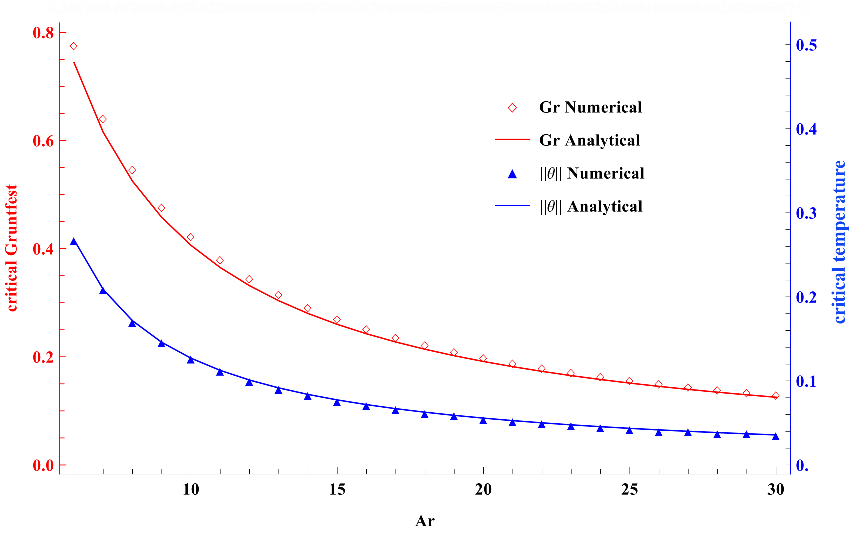

Supplementary Figure 1: **Comparison of the values of the turning point determining the onset of thermal weakening** in terms of temperature and Gruntfest number between the analytical solution (lines) and the numerical results (discrete values) as a function of the Arrhenius number.

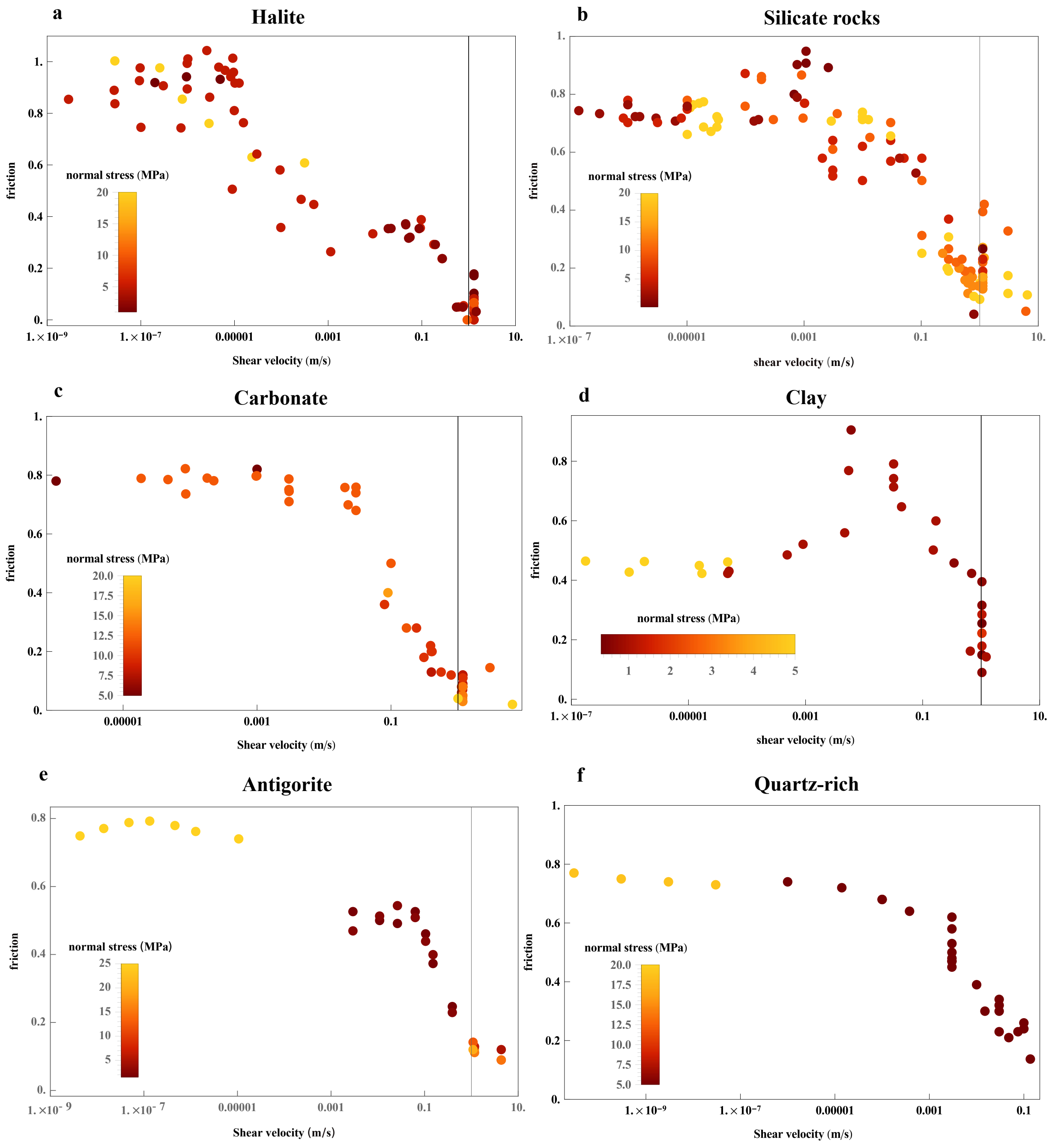

Supplementary Figure 2: **Friction evolution with the velocity and effect of normal stress for the experimental data of Fig. 3**. The colors of the different points depend on the magnitude of the normal stress applied to the bare rock or gouge material during the friction expriments for **a** halite, **b** silicate rocks, **c** carbonate rocks, **d** clay-rich rocks, **e** antigorite-rich rocks, **e** quartz-rich rocks. For this set of data, no clear dependence of the value of the friction on normal stress can be observed.

Supplementary Tables

Supplementary Table 1: **Values of the weakening coefficient for talc as a weak phase** [1,2].

Materials	Velocity (m/s)	Normal stress (MPa)	α	R^2	Reference
Antigorite/talc	$1 \cdot 10^{-7}$	100	1.5	0.997	Moore et al. 2011
Lizardite/talc	$1 \cdot 10^{-7}$	100	7.1	0.996	Moore et al. 2011
Quartz/talc	$1 \cdot 10^{-7}$	100	1.8	0.998	Moore et al. 2011
Calcite/talc	$1 \cdot 10^{-5}$	5	4.5	0.999	Giorgetti et al. 2015

Supplementary Table 2: **Values of the weakening coefficient for saturated clay as a weak phase**[3–11].

Materials	Velocity (m/s)	Normal stress (MPa)	α	R^2	Reference
Halite/muscovite	$3 \cdot 10^{-8}$	5	7	0.995	Niemeijer et al. 2005
Halite/muscovite	$1 \cdot 10^{-7}$	5	4.7	0.998	Niemeijer et al. 2005
Halite/muscovite	$1 \cdot 10^{-6}$	5	2	0.998	Niemeijer et al. 2005
Halite/muscovite	$1 \cdot 10^{-5}$	5	8.1	0.994	Niemeijer et al. 2005
Shale	$1 \cdot 10^{-6}$	10	1.7	1	Kohli et al. 2013
Quartz/Smectite	$3 \cdot 10^{-5}$	2	2.7	0.971	Oohashi et al. 2015
Quartz/Smectite	$1.5 \cdot 10^{-4}$	2	2.1	0.983	Oohashi et al. 2015
Quartz/Smectite	$1.3 \cdot 10^{-3}$	2	9.3	0.993	Oohashi et al. 2015
Montmorillonite/Quartz	$1 \cdot 10^{-6}$	40	1.6	0.995	Tembe et al. 2010
Illite/Quartz	$1 \cdot 10^{-6}$	40	$2.7 \cdot 10^{-1}$	0.999	Tembe et al. 2010
Montmorillonite/Quartz	$1.2 \cdot 10^{-6}$	75	1.6	0.996	Takahashi et al. 2007
Montmorillonite/Quartz	$1.5 \cdot 10^{-6}$	2	$6.4 \cdot 10^{-1}$	0.996	Brown et al. 2003
Montmorillonite/Quartz	$5 \cdot 10^{-7}$	50	$3.1 \cdot 10^{-2}$	0.991	Logan et al. 1987
Kaolinite/Quartz	$3 \cdot 10^{-7}$	50	$8.4 \cdot 10^{-1}$	0.998	Crawford et al. 2008
Reservoir rock	$1 \cdot 10^{-5}$	10-40	$4.8 \cdot 10^{-1}$	0.999	Zhang et al. 2019

Supplementary Table 3: **Parameters of the model inverted from the dimensionless numbers used to fit the frictional response of the different materials.** The material parameters assumed for all materials are: the thermal diffusivity $c_{th} = 0.1$mm^2/s, the exponent of the power law $m = 2$, the reference temperature $T_0 = 293°$K, the density of the strong phase $\rho_s = 2500$kg/m^3, the density of the weak phase $\rho_w = 2500$kg/m^3 (carbonate, serpentinite, halite and silicate rocks), $\rho_w = 1000$kg/m^3 (clay-rich, quartz-rich), the height of the layer is $L = 1cm$ and the height of the gouge $h = 100\mu$m. The molar masses are computed based on the standard atomic weights of the components: M(smectite)=1000g/mol, M(H_2O)=18g/mol, M(SiO_2)=60g/mol, M($CaCO_3$)=100g/mol, M(CaO)=56g/mol, M(NaCl)=58g/mol, M(Antigorite)=283g/mol, M(talc)=379g/mol.

Materials	τ_0 (MPa)	$\dot{\epsilon}^0$ (s^{-1})	Q (kJ/mol)	Qc (kJ/mol)	K_f (kJ/mol)	c_w (m^2/s)
Clay-rich	451.2	$2.8 \cdot 10^7$	58	88	$1.7 \cdot 10^{12}$	$3.6 \cdot 10^{-5}$
Halite	95.2	$3.88 \cdot 10^6$	56	93	$7.1 \cdot 10^6$	$2 \cdot 10^{-6}$
Silicate rocks	22.6	$6.82 \cdot 10^{-1}$	10	68	$4.3 \cdot 10^6$	$6.5 \cdot 10^{-7}$
Carbonate	9.5	$6.43 \cdot 10^0$	15	66	$5.6 \cdot 10^1$	$7.9 \cdot 10^{-7}$
Quartz-rich	25	$6.08 \cdot 10^0$	17	102	$7.1 \cdot 10^5$	$1.1 \cdot 10^{-6}$
Antigorite	40	$2.07 \cdot 10^5$	46	80	$2.9 \cdot 10^7$	$1.5 \cdot 10^{-5}$

Supplementary Table 4: Data for the friction coefficient as a function of the velocity for **carbonates** [12–14].

Material	normal stress (MPa)	velocity (m/s)	Residual friction	Publication
dolomite*	120	$2 \cdot 10^{-6}$	0.78	Shimamoto and Logan 1981
dolomite*	150	$2 \cdot 10^{-6}$	0.78	Shimamoto and Logan 1981
dolomite*	200	$2 \cdot 10^{-6}$	0.78	Shimamoto and Logan 1981
dolomite	75	$1 \cdot 10^{-6}$	0.58	Weeks and Tullis 1985
dolomite	75	$1 \cdot 10^{-7}$	0.56	Weeks and Tullis 1985
calcite	7.2	$4 \cdot 10^{-1}$	0.22	Han et al. 2010
calcite	7.2	$8 \cdot 10^{-2}$	0.36	Han et al. 2010
calcite	12.2	1	$7 \cdot 10^{-2}$	Han et al. 2010
calcite	4.9	1	0.12	Han et al. 2007
calcite	4.9	1	$9 \cdot 10^{-2}$	Han et al. 2007
calcite	4.9	1	0.12	Han et al. 2007
calcite	4.9	$4 \cdot 10^{-1}$	0.13	Han et al. 2007
calcite	4.9	1	0.11	Han et al. 2007
calcite	4.9	1	$7 \cdot 10^{-2}$	Han et al. 2007
calcite	6.1	1	$8 \cdot 10^{-2}$	Han et al. 2007
calcite	6.1	1	$8 \cdot 10^{-2}$	Han et al. 2007
calcite	7.2	1	$8 \cdot 10^{-2}$	Han et al. 2007
calcite	7.2	1	$6 \cdot 10^{-2}$	Han et al. 2007
calcite	7.2	$3 \cdot 10^{-1}$	0.18	Han et al. 2007
calcite	7.3	$4 \cdot 10^{-1}$	0.2	Han et al. 2007
calcite	7.3	$6 \cdot 10^{-1}$	0.13	Han et al. 2007
calcite	7.3	$8 \cdot 10^{-1}$	0.12	Han et al. 2007
calcite	7.3	1	0.11	Han et al. 2007
calcite	7.3	$2 \cdot 10^{-1}$	0.28	Han et al. 2007
calcite	7.3	$4 \cdot 10^{-1}$	0.2	Han et al. 2007
calcite	7.3	1	$5 \cdot 10^{-2}$	Han et al. 2007
calcite	7.8	1	$5 \cdot 10^{-2}$	Han et al. 2007
calcite	9.8	$2 \cdot 10^{-1}$	0.28	Han et al. 2007

Supplementary Table 5: Data for the friction coefficient as a function of the velocity for **carbonates**[14–17].

Material	normal stress (MPa)	velocity (m/s)	Residual friction	Publication
calcite	12.1	1	$5 \cdot 10^{-2}$	Han et al.. 2007
calcite	12.2	1	$8 \cdot 10^{-2}$	Han et al.. 2007
calcite	12.2	1	$8 \cdot 10^{-2}$	Han et al.. 2007
calcite	12.2	1	$4 \cdot 10^{-2}$	Han et al.. 2007
calcite	12.3	1	$5 \cdot 10^{-2}$	Han et al.. 2007
calcite	13.4	1	$3 \cdot 10^{-2}$	Han et al.. 2007
calcite	14.7	$9 \cdot 10^{-2}$	0.4	Han et al.. 2007
calcite	1.25	$1 \cdot 10^{-6}$	0.78	Di Toro et al.. unpubl.
calcite	1.25	$1 \cdot 10^{-3}$	0.82	Di Toro et al.. unpubl.
calcite*	120	$2 \cdot 10^{-6}$	0.74	Shimamoto Logan. 1981
calcite*	150	$2 \cdot 10^{-6}$	0.74	Shimamoto Logan. 1981
calcite*	200	$2 \cdot 10^{-6}$	0.74	Shimamoto Logan. 1981
calcite*	100	$5 \cdot 10^{-7}$	0.85	Morrow et al.. 2000
calcite	20	1	$4 \cdot 10^{-2}$	Spagnuolo et al. 2016
calcite	20	7	$2 \cdot 10^{-2}$	Spagnuolo et al. 2016
calcite	10	$2 \cdot 10^{-2}$	0.7	Spagnuolo et al. 2016
calcite	10	$2 \cdot 10^{-2}$	0.76	Spagnuolo et al. 2016
calcite	10	$1 \cdot 10^{-3}$	0.8	Spagnuolo et al. 2016
calcite	10	$2 \cdot 10^{-4}$	0.79	Spagnuolo et al. 2016
calcite	10	$1 \cdot 10^{-3}$	0.8	Spagnuolo et al. 2016
calcite	10	$3 \cdot 10^{-3}$	0.79	Spagnuolo et al. 2016
calcite	10	$3 \cdot 10^{-3}$	0.75	Spagnuolo et al. 2016
calcite	10	$3 \cdot 10^{-2}$	0.74	Spagnuolo et al. 2016
calcite	10	$3 \cdot 10^{-3}$	0.75	Spagnuolo et al. 2016
calcite	10	$3 \cdot 10^{-2}$	0.76	Spagnuolo et al. 2016
calcite	10	3	0.15	Spagnuolo et al. 2016
calcite	10	$1 \cdot 10^{-1}$	0.5	Spagnuolo et al. 2016
calcite	10	$3 \cdot 10^{-3}$	0.71	Spagnuolo et al. 2016

Supplementary Table 6: Data for the friction coefficient as a function of the velocity for **carbonates** [17].

Material	normal stress (MPa)	velocity (m/s)	Residual friction	Publication
calcite	10	$3 \cdot 10^{-2}$	0.68	Spagnuolo et al. 2016
calcite	10	$9 \cdot 10^{-5}$	0.74	Spagnuolo et al. 2016
calcite	10	$9 \cdot 10^{-5}$	0.82	Spagnuolo et al. 2016
calcite	10	$5 \cdot 10^{-5}$	0.79	Spagnuolo et al. 2016
calcite	10	$2 \cdot 10^{-5}$	0.79	Spagnuolo et al. 2016
calcite	10	$2 \cdot 10^{-4}$	0.78	Spagnuolo et al. 2016
calcite	10	$1 \cdot 10^{-4}$	0.78	Spagnuolo et al. 2016

Supplementary Table 7: Data for the friction coefficient as a function of the velocity for **quartz-rich rocks** [18,19].

Material	normal stress (MPa)	velocity (m/s)	Residual friction	Publication
novaculite	5	$4 \cdot 10^{-4}$	0.64	Di Toro et al.. 2004
novaculite	5	$1 \cdot 10^{-4}$	0.68	Di Toro et al.. 2004
novaculite	5	$1 \cdot 10^{-4}$	0.68	Di Toro et al.. 2004
novaculite	5	$1 \cdot 10^{-6}$	0.74	Di Toro et al.. 2004
novaculite	5	$1 \cdot 10^{-5}$	0.72	Di Toro et al.. 2004
novaculite	5	$3 \cdot 10^{-3}$	0.62	Di Toro et al.. 2004
novaculite	5	$3 \cdot 10^{-3}$	0.58	Di Toro et al.. 2004
novaculite	5	$3 \cdot 10^{-3}$	0.47	Di Toro et al.. 2004
novaculite	5	$3 \cdot 10^{-3}$	0.53	Di Toro et al.. 2004
novaculite	5	$3 \cdot 10^{-3}$	0.48	Di Toro et al.. 2004
novaculite	5	$3 \cdot 10^{-3}$	0.5	Di Toro et al.. 2004
novaculite	5	$3 \cdot 10^{-3}$	0.47	Di Toro et al.. 2004
novaculite	5	$3 \cdot 10^{-3}$	0.45	Di Toro et al.. 2004
novaculite	5	$1 \cdot 10^{-2}$	0.39	Di Toro et al.. 2004
novaculite	5	$2 \cdot 10^{-2}$	0.3	Di Toro et al.. 2004
novaculite	5	$3 \cdot 10^{-2}$	0.34	Di Toro et al.. 2004
novaculite	5	$3 \cdot 10^{-2}$	0.3	Di Toro et al.. 2004
novaculite	5	$3 \cdot 10^{-2}$	0.23	Di Toro et al.. 2004
novaculite	5	$3 \cdot 10^{-2}$	0.32	Di Toro et al.. 2004
novaculite	5	$8 \cdot 10^{-2}$	0.23	Di Toro et al.. 2004
novaculite	5	$1 \cdot 10^{-1}$	0.26	Di Toro et al.. 2004
novaculite	5	$1 \cdot 10^{-1}$	0.24	Di Toro et al.. 2004
novaculite	5	$5 \cdot 10^{-2}$	0.21	Hirose and Di Toro unpubl.
novaculite	5	$1 \cdot 10^{-1}$	0.14	Hirose and Di Toro unpubl.
sandstone	18.7	$3 \cdot 10^{-11}$	0.77	Dieterich 1979
sandstone	18.7	$3 \cdot 10^{-10}$	0.75	Dieterich 1979
sandstone	18.7	$3 \cdot 10^{-9}$	0.74	Dieterich 1979
sandstone	18.7	$3 \cdot 10^{-8}$	0.73	Dieterich 1979
sandstone	18.7	$3 \cdot 10^{-7}$	0.7	Dieterich 1979

Supplementary Table 8: Data for the friction coefficient as a function of the velocity for **silicate rocks** [18,19].

Material	normal stress (MPa)	velocity (m/s)	Residual friction	Publication
granite	2	$2 \cdot 10^{-4}$	0.71	Dieterich. 1979
granite	2	$1 \cdot 10^{-4}$	0.71	Dieterich. 1979
granite	2	$7 \cdot 10^{-6}$	0.71	Dieterich. 1979
granite	2	$3 \cdot 10^{-6}$	0.72	Dieterich. 1979
granite	2	$2 \cdot 10^{-6}$	0.72	Dieterich. 1979
granite	2	$1 \cdot 10^{-6}$	0.72	Dieterich. 1979
granite	2	$3 \cdot 10^{-7}$	0.73	Dieterich. 1979
granite	2	$1 \cdot 10^{-7}$	0.74	Dieterich. 1979
granite	5	$1 \cdot 10^{-6}$	0.78	Di Toro et al.. 2004
granite	5	$3 \cdot 10^{-3}$	0.64	Di Toro et al.. 2004
granite	5	$1 \cdot 10^{-2}$	0.62	Di Toro et al.. 2004
granite	5	$3 \cdot 10^{-2}$	0.64	Di Toro et al.. 2004
granite	5	$1 \cdot 10^{-3}$	0.77	Di Toro et al.. unpubl.
granite	5	$1 \cdot 10^{-3}$	0.77	Di Toro et al.. unpubl.
granite	5	$3 \cdot 10^{-3}$	0.52	Di Toro et al.. unpubl.
granite	5	$1 \cdot 10^{-2}$	0.5	Di Toro et al.. unpubl.
granite	5	$3 \cdot 10^{-2}$	0.57	Di Toro et al.. unpubl.
granite	5	$1 \cdot 10^{-1}$	0.58	Di Toro et al.. unpubl.
granite	5	$1 \cdot 10^{-4}$	0.87	Di Toro et al.. unpubl.
granite	5	$2 \cdot 10^{-3}$	0.58	Di Toro et al.. unpubl.
granite	5	$3 \cdot 10^{-3}$	0.54	Di Toro et al.. unpubl.
granite	5	$5 \cdot 10^{-2}$	0.58	Di Toro et al.. unpubl.
granite	10	$3 \cdot 10^{-3}$	0.61	Passelegue et al. Unpublished
granite	5	$3 \cdot 10^{-1}$	0.37	Passelegue et al. Unpublished
granite	10	$3 \cdot 10^{-1}$	0.27	Passelegue et al. Unpublished
granite	20	$3 \cdot 10^{-2}$	0.66	Passelegue et al. Unpublished
granite	20	$3 \cdot 10^{-1}$	0.31	Passelegue et al. Unpublished
granite	10	3	0.33	Passelegue et al. Unpublished

Supplementary Table 9: Data for the friction coefficient as a function of the velocity for **silicate rocks** [18–21].

Material	normal stress (MPa)	velocity (m/s)	Residual friction	Publication
granite	20	3	0.17	Passelegue et al. Unpublished
granite	20	1	0.15	Passelegue et al. Unpublished
granite	10	$1 \cdot 10^{-1}$	0.31	Passelegue et al. Unpublished
granite	20	$1 \cdot 10^{-1}$	0.25	Passelegue et al. Unpublished
granite	20	$3 \cdot 10^{-1}$	0.19	Passelegue et al. Unpublished
granite	10	$3 \cdot 10^{-1}$	0.23	Passelegue et al. Unpublished
tonalite	15	1	0.27	Di Toro et al. 2006
tonalite	10	1	0.39	Di Toro et al. 2006
tonalite	20	1	0.22	Di Toro et al. 2006
tonalite	15	1	0.24	Di Toro et al. 2006
tonalite	10	1	0.42	Di Toro et al. 2006
peridotite	15	1	0.17	Di Toro et al. 2006
peridotite	20	1	0.18	Di Toro et al. 2006
peridotite	10	1	0.22	Di Toro et al. 2006
peridotite	5	1	0.23	Di Toro et al. 2006
peridotite	13	1	0.13	Del Gaudio et al. 2009
peridotite	15.59	1	0.15	Del Gaudio et al. 2009
peridotite	16.13	1	0.16	Del Gaudio et al. 2009
peridotite	7.78	1	0.17	Del Gaudio et al. 2009
peridotite	5.37	1	0.19	Del Gaudio et al. 2009
peridotite	10.42	1	0.14	Del Gaudio et al. 2009
peridotite	12.99	1	0.14	Del Gaudio et al. 2009
peridotite	13	$8 \cdot 10^{-1}$	0.14	Del Gaudio et al. 2009
peridotite	10.4	1	0.17	Del Gaudio et al. 2009
peridotite	13	1	0.15	Del Gaudio et al. 2009
peridotite	13.01	$2 \cdot 10^{-1}$	0.25	Del Gaudio et al. 2009
peridotite	12.98	$9 \cdot 10^{-1}$	0.14	Del Gaudio et al. 2009
peridotite	13.01	$8 \cdot 10^{-1}$	0.17	Del Gaudio et al. 2009

Supplementary Table 10: Data for the friction coefficient as a function of the velocity for **silicate rocks** [17,21–23].

Material	normal stress (MPa)	velocity (m/s)	Residual friction	Publication
peridotite	13	1	0.15	Del Gaudio et al. 2009
peridotite	13.02	1	0.13	Del Gaudio et al. 2009
peridotite	10.4	$7 \cdot 10^{-1}$	0.19	Del Gaudio et al. 2009
peridotite	13	$7 \cdot 10^{-1}$	0.17	Del Gaudio et al. 2009
peridotite	13.01	$6 \cdot 10^{-1}$	0.11	Del Gaudio et al. 2009
peridotite	13.01	$6 \cdot 10^{-1}$	0.16	Del Gaudio et al. 2009
peridotite	12.98	$6 \cdot 10^{-1}$	0.15	Del Gaudio et al. 2009
peridotite	13.01	$5 \cdot 10^{-1}$	0.2	Del Gaudio et al. 2009
peridotite	10.4	$6 \cdot 10^{-1}$	0.19	Del Gaudio et al. 2009
peridotite	13	$4 \cdot 10^{-1}$	0.2	Del Gaudio et al. 2009
peridotite	10.4	$4 \cdot 10^{-1}$	0.22	Del Gaudio et al. 2009
gabbro	15.54	1	0.17	Nielsen et al. 2008
gabbro	0.13	1	0.26	Nielsen et al. 2008
gabbro	20	3	0.11	Niemeijer et al. 2011
gabbro	20	3	0.11	Niemeijer et al. 2011
gabbro	40	3	$7 \cdot 10^{-2}$	Niemeijer et al. 2011
gabbro	20	7	0.11	Niemeijer et al. 2011
gabbro	1.4	$8 \cdot 10^{-1}$	$4 \cdot 10^{-2}$	Niemeijer et al. 2011
gabbro	10	$4 \cdot 10^{-3}$	0.73	Spagnuolo et al. 2016
gabbro	10	$2 \cdot 10^{-4}$	0.86	Spagnuolo et al. 2016
gabbro	10	$9 \cdot 10^{-4}$	0.87	Spagnuolo et al. 2016
gabbro	10	$2 \cdot 10^{-4}$	0.85	Spagnuolo et al. 2016
gabbro	10	$1 \cdot 10^{-2}$	0.65	Spagnuolo et al. 2016
gabbro	10	$3 \cdot 10^{-2}$	0.7	Spagnuolo et al. 2016
gabbro	10	$1 \cdot 10^{-1}$	0.5	Spagnuolo et al. 2016
gabbro	10	$5 \cdot 10^{-1}$	0.23	Spagnuolo et al. 2016
gabbro	20	$1 \cdot 10^{-5}$	0.66	Spagnuolo et al. 2016
gabbro	20	$3 \cdot 10^{-5}$	0.67	Spagnuolo et al. 2016

Supplementary Table 11: Data for the friction coefficient as a function of the velocity for **silicate rocks** [17,24].

Material	normal stress (MPa)	velocity (m/s)	Residual friction	Publication
gabbro	20	$3 \cdot 10^{-5}$	0.69	Spagnuolo et al. 2016
gabbro	20	$3 \cdot 10^{-5}$	0.71	Spagnuolo et al. 2016
gabbro	20	$3 \cdot 10^{-5}$	0.72	Spagnuolo et al. 2016
gabbro	20	$1 \cdot 10^{-5}$	0.75	Spagnuolo et al. 2016
gabbro	20	$1 \cdot 10^{-5}$	0.76	Spagnuolo et al. 2016
gabbro	20	$1 \cdot 10^{-5}$	0.76	Spagnuolo et al. 2016
gabbro	20	$2 \cdot 10^{-5}$	0.77	Spagnuolo et al. 2016
gabbro	20	$2 \cdot 10^{-5}$	0.78	Spagnuolo et al. 2016
gabbro	20	$1 \cdot 10^{-2}$	0.74	Spagnuolo et al. 2016
gabbro	20	$2 \cdot 10^{-5}$	0.69	Spagnuolo et al. 2016
gabbro	20	$1 \cdot 10^{-2}$	0.72	Spagnuolo et al. 2016
gabbro	20	$1 \cdot 10^{-2}$	0.71	Spagnuolo et al. 2016
gabbro	20	$3 \cdot 10^{-3}$	0.71	Spagnuolo et al. 2016
gabbro	20	$1 \cdot 10^{-2}$	0.71	Spagnuolo et al. 2016
gabbro	20	$3 \cdot 10^{-1}$	0.2	Spagnuolo et al. 2016
gabbro	20	$8 \cdot 10^{-1}$	0.1	Spagnuolo et al. 2016
gabbro	20	1	$9 \cdot 10^{-2}$	Spagnuolo et al. 2016
gabbro	10	6	$5 \cdot 10^{-2}$	Spagnuolo et al. 2016
gabbro	10	$1 \cdot 10^{-5}$	0.78	Spagnuolo et al. 2016
gabbro	10	$1 \cdot 10^{-4}$	0.76	Spagnuolo et al. 2016
gabbro	10	$3 \cdot 10^{-4}$	0.71	Spagnuolo et al. 2016
gabbro	10	$9 \cdot 10^{-4}$	0.72	Spagnuolo et al. 2016
gabbro	5	$1 \cdot 10^{-6}$	0.7	Marone and Cox 1994
gabbro	5	$3 \cdot 10^{-6}$	0.7	Marone and Cox 1994
gabbro	5	$1 \cdot 10^{-5}$	0.75	Marone and Cox 1994
gabbro	5	$8 \cdot 10^{-6}$	0.72	Marone and Cox 1994
gabbro	5	$8 \cdot 10^{-7}$	0.72	Marone and Cox 1994
gabbro	2	$1 \cdot 10^{-6}$	0.77	Marone and Cox 1994

Supplementary Table 12: Data for the friction coefficient as a function of the velocity for **silicate rocks** [24,25].

Material	normal stress (MPa)	velocity (m/s)	Residual friction	Publication
gabbro	2	$1 \cdot 10^{-5}$	0.76	Marone and Cox 1994
monzodiorite	0.54	$7 \cdot 10^{-4}$	0.8	Mizoguchi Fukuyama. 2010
monzodiorite	0.54	$1 \cdot 10^{-3}$	0.91	Mizoguchi Fukuyama. 2010
monzodiorite	0.53	$3 \cdot 10^{-3}$	0.89	Mizoguchi Fukuyama. 2010
monzodiorite	1.03	$7 \cdot 10^{-4}$	0.9	Mizoguchi Fukuyama. 2010
monzodiorite	1	$1 \cdot 10^{-3}$	0.95	Mizoguchi Fukuyama. 2010
monzodiorite	2.04	$8 \cdot 10^{-4}$	0.79	Mizoguchi Fukuyama. 2010
monzodiorite	2.04	$8 \cdot 10^{-2}$	0.53	Mizoguchi Fukuyama. 2010
monzodiorite	3.04	$4 \cdot 10^{-2}$	0.58	Mizoguchi Fukuyama. 2010
monzodiorite	3.04	$9 \cdot 10^{-4}$	0.51	Mizoguchi Fukuyama. 2010

Supplementary Table 13: Data for the friction coefficient as a function of the velocity for **halite** [3,26–29].

Material	normal stress (MPa)	velocity (m/s)	Residual friction	Publication
Halite*	5	$3 \cdot 10^{-8}$	0.84	Bos et al. 2000a
Halite*	5	$9 \cdot 10^{-8}$	0.93	Bos et al. 2000a
Halite*	5	$1 \cdot 10^{-6}$	0.99	Bos et al. 2000a
Halite*	5	$9 \cdot 10^{-6}$	1.01	Bos et al. 2000a
Halite*	1	$2 \cdot 10^{-7}$	0.92	Bos et al. 2000b
Halite*	1	$9 \cdot 10^{-7}$	0.94	Bos et al. 2000b
Halite*	1	$5 \cdot 10^{-6}$	0.93	Bos et al. 2000b
Halite*	20	$3 \cdot 10^{-8}$	1	Chester 1988
Halite*	20	$3 \cdot 10^{-7}$	0.98	Chester 1988
Halite*	20	$8 \cdot 10^{-7}$	0.85	Chester 1988
Halite*	20	$3 \cdot 10^{-6}$	0.76	Chester 1988
Halite*	20	$2 \cdot 10^{-5}$	0.63	Chester 1988
Halite*	20	$3 \cdot 10^{-4}$	0.61	Chester 1988
Halite*	2	$2 \cdot 10^{-2}$	0.35	Kim et al. 2010
Halite*	2	$5 \cdot 10^{-2}$	0.37	Kim et al. 2010
Halite*	2	$5 \cdot 10^{-2}$	0.32	Kim et al. 2010
Halite*	2	$9 \cdot 10^{-2}$	0.35	Kim et al. 2010
Halite*	2	$2 \cdot 10^{-1}$	0.29	Kim et al. 2010
Halite*	2	$3 \cdot 10^{-1}$	0.24	Kim et al. 2010
Halite*	2	$5 \cdot 10^{-1}$	$4.92 \cdot 10^{-2}$	Kim et al. 2010
Halite*	2	$8 \cdot 10^{-1}$	$4.92 \cdot 10^{-2}$	Kim et al. 2010
Halite*	2	1	$3.2 \cdot 10^{-2}$	Kim et al. 2010
Halite*	5	$3 \cdot 10^{-9}$	0.85	Niemeijer 2005
Halite*	5	$3 \cdot 10^{-8}$	0.89	Niemeijer 2005
Halite*	5	$1 \cdot 10^{-7}$	0.98	Niemeijer 2005
Halite*	5	$1 \cdot 10^{-6}$	1.01	Niemeijer 2005
Halite*	5	$3 \cdot 10^{-6}$	1.04	Niemeijer 2005
Halite*	5	$5 \cdot 10^{-6}$	0.98	Niemeijer 2005

Supplementary Table 14: Data for the friction coefficient as a function of the velocity for **halite** [3,30,31].

Material	normal stress (MPa)	velocity (m/s)	Residual friction	Publication
Halite*	5	$6 \cdot 10^{-6}$	0.97	Niemeijer 2005
Halite*	5	$8 \cdot 10^{-6}$	0.94	Niemeijer 2005
Halite*	5	$1 \cdot 10^{-5}$	0.96	Niemeijer 2005
Halite*	5	$1 \cdot 10^{-5}$	0.92	Niemeijer 2005
Halite*	5	$1 \cdot 10^{-5}$	0.92	Niemeijer 2005
Halite*	5	$3 \cdot 10^{-7}$	0.91	Niemeijer 2010
Halite*	5	$1 \cdot 10^{-6}$	0.89	Niemeijer 2010
Halite*	5	$3 \cdot 10^{-6}$	0.86	Niemeijer 2010
Halite*	5	$1 \cdot 10^{-5}$	0.81	Niemeijer 2010
Halite*	5	$2 \cdot 10^{-5}$	0.76	Niemeijer 2010
Halite*	5	$3 \cdot 10^{-5}$	0.64	Niemeijer 2010
Halite*	5	$9 \cdot 10^{-5}$	0.58	Niemeijer 2010
Halite*	5	$3 \cdot 10^{-4}$	0.47	Niemeijer 2010
Halite*	5	$5 \cdot 10^{-4}$	0.45	Niemeijer 2010
Halite*	5	$1 \cdot 10^{-7}$	0.75	Buijze 2017
Halite*	5	$7 \cdot 10^{-7}$	0.74	Buijze 2017
Halite*	5	$9 \cdot 10^{-6}$	0.51	Buijze 2017
Halite*	5	$1 \cdot 10^{-4}$	0.36	Buijze 2017
Halite*	5	$1 \cdot 10^{-3}$	0.26	Buijze 2017
Halite*	5	$9 \cdot 10^{-3}$	0.33	Buijze 2017
Halite*	5	$1 \cdot 10^{-1}$	0.39	Buijze 2017

Supplementary Table 15: Data for the friction coefficient as a function of the velocity for **clay-rich gouges** [32–34].

Material	normal stress (MPa)	velocity (m/s)	Residual friction	Publication
Smectite-Quartz*	0.66	1	$9 \cdot 10^{-2}$	Mizoguchi et al. 2007
Smectite-Quartz*	0.38	1	0.15	Mizoguchi et al. 2007
Smectite-Quartz*	0.35	1	0.26	Mizoguchi et al. 2007
Smectite-Quartz*	0.64	1	0.32	Mizoguchi et al. 2007
Smectite-Quartz*	0.64	1	0.4	Mizoguchi et al. 2007
Smectite-Quartz*	1.26	1	0.29	Mizoguchi et al. 2007
Smectite-Quartz*	1.29	1	0.18	Mizoguchi et al. 2007
Smectite-Quartz*	1.87	1	0.22	Mizoguchi et al. 2007
Smectite-Quartz*	1.86	1	0.22	Mizoguchi et al. 2007
Smectite-Quartz*	0.67	$7 \cdot 10^{-1}$	0.42	Mizoguchi et al. 2009
Smectite-Quartz*	0.62	$3 \cdot 10^{-1}$	0.46	Mizoguchi et al. 2009
Smectite-Quartz*	0.62	$6 \cdot 10^{-3}$	0.91	Mizoguchi et al. 2009
Smectite-Calcite*	5	$2 \cdot 10^{-7}$	0.46	Ferri et al. 2011
Smectite-Calcite*	5	$1 \cdot 10^{-6}$	0.43	Ferri et al. 2011
Smectite-Calcite*	5	$2 \cdot 10^{-6}$	0.46	Ferri et al. 2011
Smectite-Calcite*	5	$2 \cdot 10^{-5}$	0.45	Ferri et al. 2011
Smectite-Calcite*	5	$2 \cdot 10^{-5}$	0.42	Ferri et al. 2011
Smectite-Calcite*	5	$5 \cdot 10^{-5}$	0.46	Ferri et al. 2011
Smectite-Calcite*	5	$5 \cdot 10^{-5}$	0.44	Ferri et al. 2011
Smectite-Calcite*	1	$5 \cdot 10^{-5}$	0.43	Ferri et al. 2011
Smectite-Calcite*	1	$5 \cdot 10^{-5}$	0.42	Ferri et al. 2011
Smectite-Calcite*	1	$5 \cdot 10^{-4}$	0.49	Ferri et al. 2011
Smectite-Calcite*	1	$9 \cdot 10^{-4}$	0.52	Ferri et al. 2011
Smectite-Calcite*	1	$5 \cdot 10^{-3}$	0.56	Ferri et al. 2011
Smectite-Calcite*	1	$5 \cdot 10^{-3}$	0.77	Ferri et al. 2011
Smectite-Calcite*	1	$3 \cdot 10^{-2}$	0.79	Ferri et al. 2011
Smectite-Calcite*	1	$3 \cdot 10^{-2}$	0.74	Ferri et al. 2011
Smectite-Calcite*	1	$4 \cdot 10^{-2}$	0.65	Ferri et al. 2011

Supplementary Table 16: Data for the friction coefficient as a function of the velocity for **clay-rich gouges** [34].

Material	normal stress (MPa)	velocity (m/s)	Residual friction	Publication
Smectite-Calcite*	1	$2 \cdot 10^{-1}$	0.6	Ferri et al. 2011
Smectite-Calcite*	1	$2 \cdot 10^{-1}$	0.5	Ferri et al. 2011
Smectite-Calcite*	1	$7 \cdot 10^{-1}$	0.16	Ferri et al. 2011
Smectite-Calcite*	1	1	0.14	Ferri et al. 2011
Smectite-Calcite*	1	1	$9.64 \cdot 10^{-2}$	Ferri et al. 2011
Smectite-Calcite*	1	$6 \cdot 10^{-3}$	0.89	Ferri et al. 2011
Smectite-Calcite*	1	$3 \cdot 10^{-1}$	0.46	Ferri et al. 2011
Smectite-Calcite*	1	$7 \cdot 10^{-1}$	0.42	Ferri et al. 2011
Smectite-Calcite*	1	1	0.38	Ferri et al. 2011
Smectite-Calcite*	1	1	0.3	Ferri et al. 2011
Smectite-Calcite*	1	1	0.23	Ferri et al. 2011

Supplementary Table 17: Data for the friction coefficient as a function of the velocity for **serpentinites** [35,36].

Material	normal stress (MPa)	velocity (m/s)	Residual friction	Publication
Antigorite	24.5	1	0.12	Hirose and Bystricky 2007
Antigorite	15.5	1	0.11	Hirose and Bystricky 2007
Antigorite	6.5	1	0.13	Hirose and Bystricky 2007
Antigorite	1.5	$3 \cdot 10^{-3}$	0.53	Hirose and Bystricky 2007
Antigorite	1.5	$1 \cdot 10^{-2}$	0.5	Hirose and Bystricky 2007
Antigorite	1.5	$3 \cdot 10^{-2}$	0.49	Hirose and Bystricky 2007
Antigorite	1.5	$6 \cdot 10^{-2}$	0.53	Hirose and Bystricky 2007
Antigorite	1.5	$1 \cdot 10^{-1}$	0.46	Hirose and Bystricky 2007
Antigorite	1.5	$2 \cdot 10^{-1}$	0.37	Hirose and Bystricky 2007
Antigorite	1.5	$4 \cdot 10^{-1}$	0.25	Hirose and Bystricky 2007
Antigorite	2.5	$3 \cdot 10^{-3}$	0.47	Hirose and Bystricky 2007
Antigorite	2.5	$1 \cdot 10^{-2}$	0.51	Hirose and Bystricky 2007
Antigorite	2.5	$3 \cdot 10^{-2}$	0.54	Hirose and Bystricky 2007
Antigorite	2.5	$6 \cdot 10^{-2}$	0.51	Hirose and Bystricky 2007
Antigorite	2.5	$1 \cdot 10^{-1}$	0.44	Hirose and Bystricky 2007
Antigorite	2.5	$2 \cdot 10^{-1}$	0.4	Hirose and Bystricky 2007
Antigorite	2.5	$4 \cdot 10^{-1}$	0.23	Hirose and Bystricky 2007
Antigorite	2.6	1	0.15	Hirose and Bystricky 2007
Antigorite	25	$4 \cdot 10^{-9}$	0.75	Reinen et al. 1992
Antigorite	25	$1 \cdot 10^{-8}$	0.77	Reinen et al. 1992
Antigorite	25	$5 \cdot 10^{-8}$	0.79	Reinen et al. 1992
Antigorite	25	$1 \cdot 10^{-7}$	0.79	Reinen et al. 1992
Antigorite	25	$5 \cdot 10^{-7}$	0.78	Reinen et al. 1992
Antigorite	25	$1 \cdot 10^{-6}$	0.76	Reinen et al. 1992
Antigorite	25	$1 \cdot 10^{-5}$	0.74	Reinen et al. 1992

Supplementary Table 18: Data for the friction coefficient as a function of the velocity for **serpentinites** [37].

Material	normal stress (MPa)	velocity (m/s)	Residual friction	Publication
Antigorite	5.4	1	0.14	Proctor et al. 2014
Antigorite	12.4	1	0.14	Proctor et al. 2014
Antigorite	20	1	0.12	Proctor et al. 2014
Antigorite	4.9	4	0.12	Proctor et al. 2014
Antigorite	8.8	4	$9 \cdot 10^{-2}$	Proctor et al. 2014
Antigorite	14.9	4	$9 \cdot 10^{-2}$	Proctor et al. 2014
Antigorite	19.5	4	$9 \cdot 10^{-2}$	Proctor et al. 2014

* experiments performed on gouge samples.

Supplementary Note 1

Analytical solution for the first turning point of the steady state of the system

The system of equation 8-9 in the Methods can be reduced to a one dimensional equation for sufficiently small values of the Gruntfest number and of the temperature, as the terms involving the first order chemical reaction are negligible. Following the methodology developed by Boyd[41] for the Bratu's equation, an analytical solution of the turning point, for which the temperature weakening mechanism is triggered, can be determined analytically. The simplified version of the system of equations around this turning point is:

$$\frac{\partial^2\theta}{\partial\bar{y}^2} + Gr\, e^{\frac{Ar\ \theta}{1+\theta}} = 0 \tag{1}$$

The temperature profile can be approximated by a cosine function, which respects the isothermal boundary conditions:

$$\theta(\bar{y}) = \theta_0 \cos(\pi\bar{y}) \tag{2}$$

where θ_0 is the maximum value of the temperature along the profile, chosen as the norm. This expression of the temperature profile is then replaced into equation 1 to obtain a relationship between the maximum temperature and the Gruntfest number:

$$Gr(\theta_0) = \theta_0\pi^2 e^{-\frac{Ar\ \theta_0}{1+\theta_0}} \tag{3}$$

We can then evaluate the value of θ_0 that gives the maximum Gr as a function of the Arrhenius number, corresponding to the turning point:

$$\theta_c = \frac{1}{2}(Ar - 2 - \sqrt{Ar(Ar-4)}) \tag{4}$$

The Gruntfest number for this temperature is:

$$Gr_c = \pi^2 \theta_c e^{-\frac{Ar\ \theta_c}{1+\theta_c}} \tag{5}$$

The results obtained from this analytical solution in terms of temperature θ_c and Gruntfest number Gr_c are plotted together with the numerical results obtained by pseudo-spectral methods on the full system of equations in Figure 1. The relative error between the analytical and numerical solutions obtained lies between 2 and 3.2 %.

Supplementary Note 2

Parameter inversion for the different materials

The inversion procedure relies on the fact that the response of this system of equations is self-similar in the dimensionless space, and its asymptotic response has been calculated in earlier studies[42]. Based on these studies, the inversion procedure is iterative.

First, we provide the dimensionless system with an initial guess for the values of the dimensionless groups. As common in optimization loops, the quality of the initial guess is of primary importance for the fast convergence of the loop, and for this reason we attempt to constrain the dimensionless groups as much as possible.

As a second step, using an arc-length continuation algorithm, we are iteratively optimizing the values of the dimensionless groups, to fit the experimental data (friction coefficient as a function of velocity). During this procedure, we are restricting the values of all dimensionless groups presented in the methods, and inverting for a value of the weak-phase sensitivity alpha, the reference velocity (reference strain rate times the domain size) and the reference stress, used in the mechanical law.

Finally, using the optimized values of the dimensionless groups, we are inputting the most constrained parameter values. The molar masses are calculated based on the standard atomic weights of the components. The densities of the strong phases are chosen to be 2500 kg/m^3,

representing an average value for granular materials. The densities of recrystallized halite, molten silicate rocks, nanograins of lime and talc is taken also equal to 2500 kg/m^3, but taking a more accurate value for this parameter modifies only slightly the inverted value of chemical diffusivity. Silica gel and wet illite are composed of a significant amount of water, therefore their densities is estimated to be 1000 kg/m^3. The thermal diffusivity is taken equal to 0.1 mm^2/s, which corresponds to dry granular materials[43]. From these constrained values, the least constrained parameters are inverted, namely the mass diffusivity and the kinetics (activation energy and pre-exponential factor) of the mechanics and phase transformation processes (see Table S.3).

Some values of the activation energies of phase transformation are reported in the supplementary information of [38]. For dolomite and calcite decarbonation, the values are estimated in the range of 120-130 kJ/mol. The dehydration of kaolinite has an estimated activation energy of 192 kJ/mol. Overall, it has been discussed[42] that -given all uncertainties included in estimating activation energies[44]- the activation energy Q of all mechanical processes ranges mostly from 0-60 kJ/mol, while this of phase transition processes Q_c, typically ranges between 60-300 kJ/mol. The values of Q_c we have inverted for and are summarized in Table S.3 are on the low-end of this range. However, in[38], it is also noted that the activation energy of a mechanically-activated reaction is lower than the activation energy of the same but thermally-activated reaction. Moreover, comminution tends to decrease even more the activation energy. These two effects can explain the lower values of our model compared to activation energies of the literature, which are obtained from laboratory experiments performing temperature-induced phase changes in the absence of mechanical loading.

Supplementary References

1. Moore, D. E. & Lockner, D. A. Frictional strengths of talc-serpentine and talc-quartz mixtures. *Journal of Geophysical Research: Solid Earth* **116**, 1–17 (2011).

2. Giorgetti, C., Carpenter, B. M. & Collettini, C. Frictional behavior of talc-calcite mixtures. *Journal of Geophysical Research: Solid Earth* **120**, 6614–6633 (2015).

3. Niemeijer, A. R. & Spiers, C. J. Influence of phyllosilicates on fault strength in the brittle-ductile transition: insights from rock analogue experiments. *Geological Society, London, Special Publications* **245**, 303–327 (2005). URL `http://books.google.com/books?hl=en&lr=&id=1oMGwL8ExU0C&oi=fnd&pg=PA303&c`

4. Kohli, A. H. & Zoback, M. D. Frictional properties of shale reservoir rocks. *Journal of Geophysical Research: Solid Earth* **118**, 1–17 (2013).

5. Oohashi, K., Hirose, T., Takahashi, M. & Tanikawa, W. Dynamic weakening of smectite-bearing faults at intermediate velocities: Implications for subduction zone earthquakes. *Journal of Geophysical Research : Solid Earth* **120**, 1572–1586 (2015).

6. Tembe, S., Lockner, D. A. & Wong, T. F. Effect of clay content and mineralogy on frictional sliding behavior of simulated gouges: Binary and ternary mixtures of quartz, illite, and montmorillonite. *Journal of Geophysical Research: Solid Earth* **115**, 1–22 (2010).

7. Takahashi, M., Mizoguchi, K., Kitamura, K. & Masuda, K. Effects of clay content on the frictional strength and fluid transport property of faults. *Journal of Geophysical Research: Solid Earth* **112**, 1–12 (2007).

8. Brown, K. M., Kopf, A., Underwood, M. B. & Weinberger, J. L. Compositional and fluid pressure controls on the state of stress on the Nankai subduction thrust: A weak plate boundary. *Earth and Planetary Science Letters* **214**, 589–603 (2003).

9. Logan, J. M. & Rauenzahn, K. A. Frictional dependence of gouge mixtures of quartz and montmorillonite on velocity, composition and fabric. *Tectonophysics* **144**, 87–108 (1987).

10. Crawford, B. R., Faulkner, D. R. & Rutter, E. H. Strength, porosity, and permeability development during hydrostatic and shear loading of synthetic quartz-clay fault gouge. *Journal of Geophysical Research: Solid Earth* **113**, 1–14 (2008).

11. Zhang, F., An, M., Zhang, L., Fang, Y. & Elsworth, D. The Role of Mineral Composition on the Frictional and Stability Properties of Powdered Reservoir Rocks. *Journal of Geophysical Research: Solid Earth* **124**, 1–18 (2019).

12. Shimamoto, T. & Logan, J. M. Effects of Simulated Fault Gouge on the Sliding Behavior of Tennessee Sandstone: Nonclay Gouges. *Journal of Geophysical Research* **86**, 2902–2914 (1981).

13. Weeks, J. D. & Tullis, T. E. Frictional sliding of dolomite: A variation in constitutive behavior. *Journal of Geophysical Research* **90**, 7821–7826 (1985).

14. Han, R., Shimamoto, T., Hirose, T., Ree, J. H. & Ando, J. Ultralow friction of carbonate faults caused by thermal decomposition. *Science* **316**, 878–881 (2007).

15. Han, R., Hirose, T. & Shimamoto, T. Strong velocity weakening and powder lubrication of simulated carbonate faults at seismic slip rates. *Journal of Geophysical Research: Solid Earth* **115** (2010).

16. Morrow, C. A., Moore, D. E. & Lockner, D. A. The effect of mineral bond strength and adsorbed water on fault gouge frictional strength. *Geophysical Research Letters* **27**, 815–818 (2000).

17. Spagnuolo, E., Nielsen, S., Violay, M. & Di Toro, G. An empirically based steady state friction law and implications for fault stability. *Geophysical Research Letters* **43**, 3263–3271 (2016).

18. Di Toro, G., Goldsby, D. L. & Tullis, T. E. Friction falls towards zero in quartz rock as slip velocity approaches seismic rates. *Nature* **427**, 436–439 (2004).

19. Dieterich, J. H. Modeling of rock friction: 1. Experimental results and constitutive equations. *Journal of Geophysical Research* **84**, 2161 (1979). URL `http://doi.wiley.com/10.1029/JB084iB05p02161`.

20. Di Toro, G., Hirose, T., Nielsen, S., Pennacchioni, G. & Shimamoto, T. Natural and experimental evidence for fault lubrication during earthquakes. *Science* **311**, 648–649 (2006).

21. Del Gaudio, P. *et al.* Frictional melting of peridotite and seismic slip. *Journal of Geophysical Research* **114** (2009).

22. Nielsen, S., Di Toro, G., Hirose, T. & Shimamoto, T. Frictional melt and seismic slip. *Journal of Geophysical Research: Solid Earth* **113**, 1–20 (2008).

23. Niemeijer, A., Di Toro, G., Nielsen, S. & Di Felice, F. Frictional melting of gabbro under extreme experimental conditions of normal stress, acceleration, and sliding velocity. *Journal of Geophysical Research: Solid Earth* **116**, 1–18 (2011).

24. Marone, C. J. & Cox, S. J. D. Scaling of Rock Friction Constitutive Parameters: The Effects of Surface Roughness and Cumulative Offset on Friction of Gabbro. *Pure and Applied Geophysics* **143**, 359–385 (1994).

25. Mizoguchi, K. & Fukuyama, E. Laboratory measurements of rock friction at subseismic slip velocities. *International Journal of Rock Mechanics and Mining Sciences* **47**, 1363–1371 (2010).

26. Bos, B., Peach, C. J. & Spiers, C. J. Frictional-viscous flow of simulated fault gouge caused by the combined effects of phyllosilicates and pressure solution. *Tectonophysics* **327**, 173–174 (2000).

27. Bos, B., Peach, C. J. & Spiers, C. J. Slip behavior of simulated gouge-bearing faults under conditions favoring pressure solution. *Journal of Geophysical Research: Solid Earth* **105**, 16699–16717 (2000).

28. Chester, F. M. The brittle-ductile transition in a deformation-mechanism map for halite. *Tectonophysics* **154**, 125–136 (1988).

29. Kim, J. W., Ree, J. H., Han, R. & Shimamoto, T. Experimental evidence for the simultaneous formation of pseudotachylyte and mylonite in the brittle regime. *Geology* **38**, 1143–1146 (2010).

30. Niemeijer, A., Marone, C. & Elsworth, D. Frictional strength and strain weakening in simulated fault gouge: Competition between geometrical weakening and chemical strengthening. *Journal of Geophysical Research: Solid Earth* **115**, 1–16 (2010).

31. Buijze, L., Niemeijer, A. R., Han, R., Shimamoto, T. & Spiers, C. J. Friction properties and deformation mechanisms of halite(-mica) gouges from low to high sliding velocities. *Earth and Planetary Science Letters* **458**, 107–119 (2017). URL `http://dx.doi.org/10.1016/j.epsl.2016.09.059`.

32. Mizoguchi, K., Hirose, T., Shimamoto, T. & Fukuyama, E. Reconstruction of seismic faulting by high-velocity friction experiments: An example of the 1995 Kobe earthquake. *Geophysical Research Letters* **34** (2007).

33. Mizoguchi, K., Hirose, T., Shimamoto, T. & Fukuyama, E. High-velocity frictional behavior and microstructure evolution of fault gouge obtained from Nojima fault, southwest Japan. *Tectonophysics* **471**, 285–296 (2009).

34. Ferri, F. *et al.* Low- to high-velocity frictional properties of the clay-rich gouges from the slipping zone of the 1963 Vaiont slide, northern Italy. *Journal of Geophysical Research: Solid Earth* **116**, 1–17 (2011).

35. Hirose, T. & Bystricky, M. Extreme dynamic weakening of faults during dehydration by coseismic shear heating. *Geophysical Research Letters* **34**, 10–14 (2007).

36. Reinen, L. A., Tullis, T. E. & Weeks, J. D. Two-Mechanism Model for Frichonal Sliding of Serpentinite. *Geophysical Research Letters* **19**, 1535–1538 (1992).

37. Proctor, B. P. *et al.* Dynamic weakening of serpentinite gouges and bare surfaces at seismic slip rates. *Journal of Geophysical Research : Solid Earth* **119**, 8107–8131 (2014).

38. Di Toro, G. *et al.* Fault lubrication during earthquakes. *Nature* **471**, 494–498 (2011). URL `http://www.nature.com/doifinder/10.1038/nature09838`.

39. Kim, J. W., Ree, J. H., Han, R. & Shimamoto, T. Experimental evidence for the simultaneous formation of pseudotachylyte and mylonite in the brittle regime. *Geology* **38**, 1143–1146 (2010).

40. Tesei, T., Harbord, C. W., De Paola, N., Collettini, C. & Viti, C. Friction of Mineralogically Controlled Serpentinites and Implications for Fault Weakness. *Journal of Geophysical Research: Solid Earth* **123**, 6976–6991 (2018).

41. Boyd, J. P. An analytical and numerical study of the two-dimensional Bratu equation. *Journal of Scientific Computing* **1**, 183–206 (1986).

42. Alevizos, S., Poulet, T. & Veveakis, M. Thermo-poro-mechanics of chemically active creeping faults. 1: Theory and steady state considerations. *Journal of Geophysical Research: Solid Earth* n/a–n/a (2014).

43. Yun, T. S. & Santamarina, J. C. Fundamental study of thermal conduction in dry soils. *Granular Matter* **10**, 197–207 (2008).

44. L'vov, B. V. Thermal Decomposition of Solids and Melts New Thermochemical Approach to the Mechanism, Kinetics and Methodology. In Simon, J. (ed.) *Hot Topics in Thermal Analysis and Calorimetry*, 263 (Springer, Dordrecht, Netherlands, 2007).
